# Willingness to Participate in Hypothetical HIV Vaccine Trial and Associated Factors among People Who Inject Drugs in Dar es Salaam, Tanzania

**DOI:** 10.1155/2020/8507981

**Published:** 2020-07-02

**Authors:** Masunga K. Iseselo, Edith A. M. Tarimo, Eric Sandstrom, Asli Kulane

**Affiliations:** ^1^Department of Clinical Nursing, Muhimbili University of Health and Allied Sciences, Dar es Salaam, Tanzania; ^2^Department of Nursing Management, Muhimbili University of Health and Allied Sciences, Dar es Salaam, Tanzania; ^3^Department of Clinical Science and Education, Södersjukhuset, Karolinska Institutet, Stockholm, Sweden; ^4^Equity and Health Policy Research Group, Department of Public Health Sciences, Karolinska Institutet, Stockholm, Sweden

## Abstract

This study is aimed at assessing the willingness to participate in the HIV vaccine trials and the associated factors among people who inject drugs (PWIDs) in Tanzania. Information about the willingness to participate and the associated factors was collected using interviewer-administered questionnaires at the medication treatment for opioid use disorder (MOUD) clinic in Dar es Salaam. Data analysis was performed using the IBM SPSS Statistic 20. The mean age of respondents was 36.7, and the standard deviation (SD) was ±7.2. The majority of respondents (68%) had primary education, and a high proportion of them were single (61.5%). More than one-third (37.9%) shared needles and syringes. Most (87.3%) had more than three sexual partners, and almost half (51.4%) did not use condoms during sexual intercourse with nonregular partners. About 63% had knowledge of HIV transmission while 27% had heard about HIV vaccine trials. Generally, 76% of the respondents expressed willingness to participate in future HIV vaccine trials regardless of prior knowledge of HIV vaccine trials. Willingness to participate in HIV vaccine trials was not associated with education level, people living with, knowledge about HIV transmission, awareness of HIV vaccine trials, sharing of syringe/needles, and number of sexual partners. Only older age (OR = 1.6, 95%CI = 1.01, 2.6) and condom use (OR = 0.49, 95%CI = 0.26, 0.97) showed an association with willingness. However, after performing logistic regression with factors at *p* value ≤ 0.2 to ascertain the other factors on the effects of age, condom use, education level, and sharing of needles/syringes, the results were not statistically significant. Although participants reported a high willingness to participate in hypothetical HIV vaccine trials, no definitive conclusion can be drawn about the associated factors. Further studies with intensive educational programs are needed to investigate the factors on willingness to participate in actual HIV vaccine trials among PWIDs.

## 1. Introduction

In 2017, 36.8 million people were living with HIV of which 1.9 million people became newly infected globally. Sub-Saharan Africa is the most affected region, with 19.6 million people living with HIV and accounts for more than half of the global total HIV infections [1]. Tanzania is among the countries in sub-Saharan Africa hit by the HIV epidemic impacting both the economic and social sectors. On average, 4.9% of adults are infected with HIV in Tanzania, the prevalence being almost twice as high among women (6.3%) as compared to men (3.4%) [2].

Despite the current HIV prevalence, Tanzania has done well to control the HIV infection over the last decade. Increasing access to antiretroviral treatment has helped Tanzania decrease the impact of the epidemic. Consequently, between 2010 and 2015, the number of new infections declined by more than 20% and the number of people dying from an AIDS-related illness halved [3].

The HIV infection rate among key populations such as commercial sex workers (CSW), men who have sex with men (MSM), and PWIDs is 2-10 times higher than that of the general population [4]. PWIDs are particularly vulnerable to HIV and other blood-borne pathogens. This could be the result of sharing contaminated syringes and other injecting equipment compounded by a lack of knowledge regarding HIV and low HIV testing levels [5–9]. It is estimated that there are 30,000 PWIDs in mainland Tanzania [10]. Different harm reduction strategies have been implemented to tackle this problem. This includes medication treatment for opioid use disorder (MOUD), needle and syringe exchange programs (NSP), and sober houses for residential treatment in Dar es Salaam and Zanzibar [11]. A systematic review examining the coverage of needle and syringe exchange program in low and middle-income countries shows that needle and syringe exchange program (NSP) is effective in the reduction of HIV infection among PWIDs [12]. As has been reported more than a decade ago, the best hope for halting the global HIV epidemic is the development of an effective, safe, and affordable preventive vaccine [13]. Clinical trials for HIV vaccines include three distinct phases: phase I/II trials are conducted on a small to moderate numbers of healthy participants at low risk for HIV infection to assess the safety and immunogenicity of the vaccine candidate [14]. A range of sociobehavioural issues needs to be addressed before moving to the next phase of the vaccine trials. Phase III HIV vaccine trials (or efficacy trials) are conducted on a large scale, with participants at high risk for HIV infection defined as an HIV incidence rate of 2% or more [15].

The involvement of high-risk populations such as PWIDs in HIV vaccine efficacy trials is important because they are the target population for an effective preventive HIV vaccine. Assessment of willingness to participate in HIV/AIDS studies is significant and addresses the suitability of populations for potential HIV vaccine efficacy trials. Studies in low-income countries reported factors associated with willingness to participate in HIV vaccine trials in different population groups [16–18]. The previous phase I/II HIV vaccine trials involving low-risk participants in Dar es Salaam [19, 20] are the stepping stone for the preparation of HIV vaccine efficacy trials which involves high-risk populations such as PWIDs.

Studies assessing prevention interventions, including prophylactic vaccines, require the recruitment of high-risk volunteers who are willing to participate in vaccine trials [21, 22]. Previous studies in different countries revealed moderate to a high level of willingness to participate in HIV vaccine trials among PWIDs [23, 24]. Several factors can influence the willingness to participate in HIV vaccine trials among study participants. For example, a study in Canada with HIV-negative participants reported that self-efficacy was positively related to willingness to participate in HIV vaccine trials while HIV treatment optimism and knowledge of vaccine trial concepts were unrelated to the willingness to participate [25]. Recruiting high-risk populations to participate in HIV vaccine trials is a critical feature of vaccine preparedness studies designed to assess the feasibility of conducting HIV vaccine efficacy trials. As reported elsewhere, the high-risk population poses multiple challenges such as retention in the study and the number of visits in actual trials [18]. There is a paucity of empirical data on willingness to participate in HIV vaccine trials among PWIDs and the associated factors in Tanzania. To gain knowledge on this aspect, we assessed the willingness to participate in the hypothetical HIV vaccine trial and the associated factors among HIV-negative PWIDs in Dar es Salaam.

## 2. Materials and Methods

### 2.1. Design

We adopted a cross-sectional descriptive study design [26]. The design was carefully chosen to determine the willingness to participate in HIV vaccine trial and the associated sociobehavioural characteristics among PWIDs. Moreover, the design was appropriate for ascertaining the risk behaviour of the participants for planning future HIV vaccine efficacy trials.

### 2.2. Setting

This study was conducted at the medication treatment for opioid use disorder (MOUD) clinic, Muhimbili National Hospital (MNH) in Dar es Salaam, Tanzania. The site was one of the three MOUD clinics providing methadone to the PWIDs in the region. Also, the clinics provided other services such as psychosocial support, screening, and testing for HIV infection, Tuberculosis (TB), and other diseases potentially transmitted by the sharing of needles or syringes. Urine screening and counseling were the mandatory services in the clinic. MNH clinic provided services to more than 2000 PWIDs who came for their daily maintenance doses.

### 2.3. Population and Participants

The study participants were HIV-negative PWIDs. We used the clinic records to determine the potential respondents' HIV status. We aimed for PWIDs who were attending at MOUD clinic due to their availability. Previous research in the United States (US) revealed several challenges in recruiting PWIDs from the community [27]. In this case, using participants at the MOUD clinic helped to overcome some of the challenges observed in the US and this facilitated getting the appropriate participants with low drug withdrawal effects that could hinder cooperation. Both men and women were included in the study.

### 2.4. Sample Size Estimation

Initially, we calculated a sample size of 423 study participants. We used the following formula:

(1) $$\begin{array}{c} N=\frac{Z^{2}P\left(1-P\right)}{d^{2}}, \end{array}$$

where *N* is the sample size, *Z* is the *Z* value for a level of confidence, e.g., 1.96 (95%), *P* is the expected proportion or prevalence of 0.5 (unknown proportion), and *d* is the confidence interval = 0.05.

(2) $$\begin{array}{c} N=\frac{{\left(1.96\right)}^{2}\times 0.5\left(1-0.5\right)}{0.05\times 0.05}=384+10\%\left(\text{nonresponse rate}\right)=423. \end{array}$$

### 2.5. Sampling Procedure

We used probability sampling methods [28] in which a simple random sampling procedure was employed to select the respondents. We assigned numbers to clients in a list of 1200 PWIDs from the MOUD clinic records as a sampling frame. We then used a calculator to randomly select from a list without replacements of the potential respondents who came to the clinic for their daily doses. The contact person informed the eligible respondents about the study. When the client agreed, he/she was directed to see the research assistants/researcher for consent and data collection. The list was updated every day to remove those participants who had already participated. Participants were given USD 0.85 (Tshs 2000) as compensation for their time and transport.

### 2.6. Inclusion and Exclusion Criteria

It is worthwhile to note that we specifically included respondents who were stable, clinically healthy, and HIV negative. Respondents with medical conditions such as TB, Hepatitis (B and C), and HIV-positive and those who were weak and drowsy due to drug use were excluded from the study.

### 2.7. Data Collection

#### 2.7.1. Data Collection Tool

An interviewer-administered questionnaire was used to collect the data from June to September 2018. We modified the questionnaire used in the previous studies in Tanzania [19, 20] by adding some items related to injecting drug behaviours. The questionnaire was prepared in the English language and then translated into the Swahili language by using standard translating procedures [29, 30]. The data collection tool consisted of 8 questions on sociodemographic characteristics, 13 questions on injecting the drug, and risky sexual behaviours. Furthermore, the questionnaire consisted of eight (8) yes/no questions that assessed knowledge about HIV transmission and HIV vaccine trials. One “yes or no” question assessed the willingness to participate in HIV vaccine trial.

#### 2.7.2. Validity and Reliability

Two psychiatrists and one mental health nurse working in the methadone at MNH clinic reviewed the questionnaire and provided inputs that improved the content and face validity of the tool. In addition, a former injecting drug user, who was also a peer educator, reviewed the questionnaire for the improvement of language common in PWIDs. To ensure clarity and objectivity of the questions, we piloted the questionnaire with 40 respondents as guided by Perneger et al. [31]. The internal consistency was assessed by using Cronbach's alpha (*α* = 0.74) which was found to be in an acceptable range [32]. The results of the pilot helped to refine the questionnaire by revising the heterogeneous constructs of the question items. Data from the pilot study were not included in the final analysis. As the consistency and validity of the study instrument were stabilized, it was made available for data collection. Also, the first author trained the research assistant about the study purpose and the procedure before data collection and monitored the data collection process throughout the study period. This also ensured the quality of the data collected.

#### 2.7.3. Participants' Briefing Sessions

Before the commencement of data collection, we briefly described the conduct of HIV vaccine trials to participants. The briefing sessions took place on the hospital premises. The sessions included the mechanism of action of candidate vaccines, how it would be administered, what to expect if the participant is enrolled in the study, the issues related to vaccine-induced seropositivity and how they would be handled, and the absence of assured protection and the potential for social and physical harm to the participants. The purpose of the briefing session was to allow the participants to understand what experimental vaccines are and how they are being used.

### 2.8. Outcome Variables

The outcome variable of the study was the willingness to participate in HIV vaccine trial. The predictor variables were the age of participants, the person living with, the level of education, knowledge about HIV Transmission, awareness of HIV vaccine trials, sharing of syringe/needle, and number of sexual partners.

### 2.9. Data Processing and Analysis

The analysis of the data took place in two stages. We applied univariate analysis in terms of frequency distributions to describe the study participants, the dependent and independent variables. All variables were assessed as categorical and were expressed as percentages except age at onset of injecting drugs, which was analyzed as a continuous variable. Eight statements assessed knowledge on HIV/AIDS transmission where each correct answer scored 1 and incorrect one scored 0. The scores of knowledge of HIV/AIDS transmissions were further categorized as “not knowledgeable” for those who scored <4 and “knowledgeable” for those who scored >4 using a modified Bloom cut-off point as reported in another study [33]. Knowledge scores for individuals were calculated and summed up to give the total knowledge score. Awareness about the HIV vaccine trial was assessed by asking if respondents had ever heard about HIV vaccine trials before the current study. Answers were dichotomized into yes/no fashion. However, those who answered yes were further asked to qualify their source of information: whether they heard from the media, health workers, friends, or relatives.

Bivariate analysis was used to examine the association between willingness to participate in HIV vaccine trial and sociodemographic characteristics, knowledge of HIV transmission, awareness of HIV vaccine trials, and HIV risk behaviours. Odds ratio (OR) and 95% CIs were used to determine the association between willingness to participate in HIV vaccine trials and independent categorical variables. We performed binary logistic regression analyses to determine the effect size of the independent variables over the willingness to participate in HIV vaccine trial. The effect size was compared to the *p* value < 0.05. Missing values were not analyzed, and nonsignificant results were disregarded. All analyses were performed using the IBM SPSS Statistic 20.

### 2.10. Ethical Approval

We obtained the ethical clearance from the Institutional Review Board of Muhimbili University of Health and Allied Sciences (MUHAS) with Ref. No. 2017-06-28/AEC/Vol.XII/85. Further, permission to conduct the study was obtained from MNH. We explained the aim, nature, benefit, and risk of the study to the participants. Moreover, the right to withdraw from the study was clearly described. We ensured the anonymity of the information provided by using codes instead of their names in all documents. Written informed consent was obtained from all participants before data collection.

## 3. Results

### 3.1. Sociodemographic Characteristics of Respondents

We recruited 386 respondents resulting in 91.3% of the intended sample size. We did not reach 100% of the sample size because some respondents were excluded from the study because of seropositivity while others defaulted or absconded from the MOUD program for unknown reasons. The age of respondents ranged between 18 and 58 years with a mean age of 36.7 and a standard deviation (SD) of ±7.2, and more than a half (52.1%) were between 36 and 58 years. Nearly all of the respondents (96%) were male. Most were single (61.5%), which included those who were separated, divorced, or widowed. One-third of the respondents lived with their parents, 68% had attained primary education, and almost all (93.5%) were self-employed. i.e., working in day-labour jobs such as gardening, car wash, and petty traders (Table 1).

### 3.2. Risk Behaviour

The mean age of onset for injecting drugs was 23.6 (SD ± 6.1). More than one-third of the respondents shared needles or syringes of whom 41.1% had shared with three or more friends. Almost 70% had shared needle and syringe with other people for more than 3 times in their lifetime. Of those who had shared needles/syringes, 83.2% tried to clean the syringes/needles using cold water. Most (87.3%) of respondents had more than three sexual partners in their lifetime. Almost a half (47.7%) had had sexual intercourse in the last 6 months with someone other than a regular partner. Slightly more than half (51.4%) of respondents who had sexual intercourse did not use condoms with the other partners. A third (31.7%) of the partners were injecting drugs. Many (43.0%) had ever exchanged sex with money, drugs, or other services that increased their likelihood of HIV infection (Table 2).

### 3.3. Knowledge of HIV Transmission and HIV Vaccine Trials

Almost all (99.5%) reported unprotected sex as the principal method of HIV transmission. The majority of the respondents (99%) thought that sharing of needles was the main method of HIV infection followed by mother to child transmission (84%) and during delivery (80%), respectively. A small proportion (14.2%) of the respondents reported that they can recognize a person living with HIV through physical appearance. Only 35.5% reported a difference between HIV and AIDS. It was reported that 27.5% had heard about HIV vaccine trials of whom 45.3% heard from health care workers. About 62% were knowledgeable about HIV transmission (Table 3).

### 3.4. Willingness to Participate in Future HIV Vaccine Trial and Its Associated Factors

Seventy-six percent of the respondents would participate in future HIV vaccine trials if given a chance. Willingness to participate was not associated with the person living with, level of education, knowledge of HIV transmission, and awareness of HIV vaccine trials. Furthermore, willingness to participate was not statistically associated with the sharing of needle/syringe, the number of sexual partners, sexual partners who injects drug or who exchanges sex, and other commodities. Most factors had 95% CI spanning through 1. Only the older age (OR = 1.6, 95%CI = 1.01, 2.6) and condom use (OR = 0.49, 95%CI = 0.26, 0.97) were statistically associated with willingness to participate in future HIV vaccine trials. However, logistic regression was performed with factors at *p* value ≤ 0.2 to ascertain the other factors on the effects of age, education level, sharing of needles/syringes, and condom use. The results were not statistically significant (Table 4).

## 4. Discussion

This is the first study in Tanzania that assesses willingness to participate in a hypothetical HIV vaccine trial and its associated factors among PWIDs. Overall, more than three quarters of respondents are willing to participate in future HIV vaccine efficacy trials. The study informs no association between willingness to participate in hypothetical HIV vaccine trials with knowledge of AIDS/HIV transmission, awareness of HIV vaccine trial, level of education, and number of sexual partners. Also, our study has shown that living with other people and sharing of needles/syringes are not statistically significant factors for the respondents to participate in the trials. Our study did not find any important factors that could be associated with willingness to participate in HIV vaccine trials except the old age and condom use which could be the effects of confounders. The high prevalence of respondents willing to participate in HIV vaccine trials implies that people who inject drugs are potential participants in the actual trial. Therefore, this study gives information that can be used in the planning and recruitment of participants in more controlled vaccine trials for a similar population.

### 4.1. Willingness to Participate in HIV Vaccine Trials and Sociodemographic Characteristics

Our study revealed that the majority of respondents had attained primary education level or lower. This may be caused by early drop out from schools due to substance use, a common problem in Tanzania [34]. The evidence is also reported elsewhere [35, 36]. A low level of education among participants may be a stumbling block to obtain HIV-related information. It is known that a high level of education facilitates access to information and better understandings of HIV transmission and prevention [37–39]. In addition, a high level of education increases individuals' risk perception towards HIV infection which may have an impact on willingness to participate in HIV vaccine trials. Though a high proportion of participants in our study had primary education, the notion of willingness to participate is not conclusive and therefore needs further investigation.

The results of our study have also shown that willingness to participate in HIV vaccine trials is weakly associated with older age. No definitive explanations for this relationship can be elicited from our study. The possible explanation may be due to the third variables that were not assessed. In HIV vaccine trials, age is an important factor to be considered when recruiting study participants. For a high-risk efficacy trial, young age is more preferred compared to 18-40 years for immunogenicity studies. Studies in other countries revealed strong relationships between young age (20-35 years) and willingness to participate in HIV vaccine trials [40–43]. Although we can not conclude based on these results that older age is a predictor for willingness to participate in HIV vaccine trials, more researches are needed that focus on other factors that affect ages.

Even though the majority of respondents in our study were living with relatives, close friends, or spouses, no association is found with the willingness to participate in HIV vaccine trials. It is a common practice people to share information in decision-making, especially with close people. The current study has shown that participants' willingness to participate in an HIV vaccine trial is independent of the person they are living with or close friends. Other studies in Dar es Salaam reported living with people influences decision-making toward participation in the HIV vaccine trial [19, 20]. In other countries, family support was associated with willingness to participate in HIV vaccine trials among people who use drugs [23, 44].

### 4.2. Risk Behaviour and Willingness to Participate in HIV Vaccine Trials

Our study revealed that the sharing of needles and syringes was a common practice among PWIDs. Despite the high knowledge of participants about the transmission of HIV through sharing syringes, the issues of cost, stigma, and fear of police are the major barrier for obtaining purchasing new syringes. The fact that needle and syringe sharing practices had no association with willingness to participate in HIV vaccine trials in the current study can be described by the experience of the respondents being exposed to harm reduction activities before joining the MOUD program at MNH. More details of the harm reduction are reported elsewhere [11]. This might have increased the awareness of different HIV prevention programs including HIV vaccine trials among PWIDs. Our findings differ from the studies in high-income countries that reported frequent needle exchange attendance [24] and sharing of needle/syringes [23, 45] were associated with willingness to participate in HIV vaccine trials. The difference may be accounted for either by differences in the study setting or by the socioeconomic factors of the participants from those countries and Tanzania.

Respondents in our study had multiple sexual partners. Even though the majority were willing to participate in the hypothetical HIV vaccine studies, the number of sexual partners has no association with willingness to participate. However, the use of condoms among participants was relatively associated with willingness to participate. We cannot rely on this association as it may be due to unknown factors. In contrast, studies in China and Spain revealed a positive association with willingness to participate in HIV vaccine trials among multiple sexual partners with other drug users or commercial sex workers [23, 46]. This implies that multiple sexual partners increase the perception of increased risk of HIV infection and hence prompt the individual for participation in the trials.

### 4.3. Knowledge of HIV Transmission and Awareness of HIV Vaccine Trial

The high knowledge of HIV transmission can be described by the fact that participants were recruited from the MOUD clinic which conducts health education sessions about HIV transmission and preventions. The level of knowledge might be reflected differently if participants were recruited from the community. This makes our finding inconclusive. Contrary to our results, a study in Bangladesh revealed a low level of knowledge on HIV transmission among PWIDs [47]. The reason for the difference may be attributed to the exposure to the HIV prevention program offered at the MOUD clinic among participants in our study. Although participants were knowledgeable about HIV transmission, we found no association with willingness to participate in hypothetical HIV vaccine trials. The reason for this lack of association is not clear and hence needs further investigations. In the current study, the willingness to participate is high. This indicates that, regardless of the prior knowledge of and awareness of HIV vaccine trials, participants may still be willing to participate. Similar findings were reported in Canada for both HIV-negative and HIV-positive PWIDs [25, 48]. It is also important to note that there is a need for education about HIV vaccine trials to enhance participants' understanding and knowledge in their decision-making process as reported elsewhere [49].

The low level of awareness of HIV vaccine trials in our study implies little effort has been done to disseminate information related to HIV vaccine research among PWIDs. Although health care providers were mentioned as the source of information, it was not assessed whether MOUD providers or elsewhere was the source of information.

### 4.4. Limitation

This study may be limited by the use of interviewer-administered questionnaires. This might influence the response leading to the overestimation of the willingness to participate in our study sample. However, the questionnaire was constructed in such a way that it had enough options and minimal question order bias. Also, our study assessed willingness to participate in hypothetical vaccine trials. There may be a gap between expressed willingness and actual participation decision. More studies are needed to assess the actual participation in HIV vaccine trials among PWIDs in Tanzania. Our study also recruited participants from the MOUD clinic that might not reflect the PWIDs in the community who are not on methadone treatment. However, careful selection of the study sample and timing of the data collection enabled obtaining the information that is useful for planning future HIV vaccine trial.

## 5. Conclusions

The majority of the respondents were willing to participate in HIV vaccine trials regardless of prior knowledge and information about HIV vaccine trials. The level of education and type of person living with had no relationship with willingness to participate in the trials. Similarly, sharing of needles/syringes, the number of sexual partners, and the level of knowledge on HIV transmission had no significant association towards the willingness to participate in HIV vaccine trials. Further studies with intensive educational programs are needed to investigate the willingness to participate in actual HIV vaccine trials among PWIDs.

## Figures and Tables

**Table 1:** Sociodemographic characteristics of respondents.

Sociodemographic characteristics	Number (%), *N* = 386
Age (mean = 36.7, SD = 7.2) years	
Young adults (18-35)	183 (47.4)
Adults (36-58)	203 (52.6)
Gender	
Male	370 (95.9)
Female	16 (4.1)
Marital status	
Single^a^	236 (61.5)
Married	148 (38.5)
Level of education	
Primary school or lower	273 (70.7)
Secondary school or higher	113 (29.3)
People living with	
Parents	134 (34.7)
Significant others^b^	252 (65.3)
Occupation	
Self-employed^c^	359 (93.5)
Formally employed	25 (6.5)

^a^Single includes single, separated, divorced, and widowed. ^b^Relatives, close friends, and spouse. ^c^Day-labour work including gardening, car wash, petty traders, and directed towards the acquisition of money for subsistence.

**Table 2:** Risk behaviours of respondents.

Behaviour	Number (%), *N* = 386
Age at onset of injecting drugs (mean = 23.6, SD = 6.1) years	
Shared needle/syringes	
Yes	146 (37.9)
No or do not remember	240 (62.1)
Number of people shared needle/syringes	
One or two	86 (58.9)
Three or more	60 (41.1)
Frequency of needle or syringe sharing with other people	
One or two times	46 (31.0)
Three times or above	100 (69.0)
Types of agents used to clean needle/syringe before sharing	
Coldwater	109 (83.2)
Others^d^	22 (16.8)
Number of sexual partners	
One or two	49 (12.7)
Three or more	337 (87.3)
Had sexual intercourse with nonregular partner in the last 6 months	
Yes	184 (47.7)
No or do not remember	202 (52.3)
Used condom with nonregular partners in the last intercourse	
Yes	89 (48.6)
No or do not remember	94 (51.4)
Partner injecting drugs	
Yes	63 (31.7)
No	136 (68.3)
Exchanged sex for money, drugs, or other services	
Yes	166 (43.0)
No	220 (57.0)

SD: standard deviation. ^d^Jik, hot water, spirit, and dry cloth.

**Table 3:** Knowledge of HIV transmission and HIV vaccine trials.

Item	Number (%), *N* = 386
How is HIV transmitted?	
Sharing of sharp instruments such as syringes	382 (99.0)
Mother to child transmission through breastfeeding	323 (83.7)
Mother to child transmission through delivery	308 (79.8)
Unprotected sex with an HIV-infected person	378 (97.9)
Can you recognize a person with AIDS by physical appearance?	
Yes	55 (14.2)
No	331 (85.8)
Is there a difference between a person with HIV infection and AIDS	
Yes	137 (35.5)
No	249 (64.5)
Have you ever heard about HIV vaccine trials before this study?	
Yes	106 (27.5)
No	280 (72.5)
Source of information about HIV vaccine trials	
Health care workers	48 (45.3)
Friends	38 (35.8)
Media	20 (18.9)
Knowledge about HIV transmission	
Knowledgeable	242 (62.7)
Not knowledgeable	144 (37.3)

**Table 4:** Willingness to participate in HIV vaccine trials and its associated factors.

Respondents	Willing to participate 294 (76.2%)		
Factors	Number (%) willing	Odds ratio (95% CI)	
		Unadjusted	Adjusted
Age (groups)			
18-35	131 (71.6)	1	
36-58	163 (80.3)	1.6 (1.01, 2.6), *p* < 05	0.89 (.45, 1.748), *p* = 0.736
Person living with			
Parents	105 (78.4)	1	
Significant others	189 (75.0)	0.8 (0.5, 1.4)	
Level of education			
Primary or lower	202 (74.0)	1.5 (0.9, 2.7), *p* = 0.1	0.59 (0.26, 1.35), *p* = 0.21
Secondary or higher	92 (81.4)	1	
Knowledge about HIV transmission			
Knowledgeable	185 (76.4)	1.0 (.6, 1.6)	
Not knowledge	109 (75.7)	1	
Awareness of HIV vaccine trials			
Yes	211 (75.4)	1.2 (0.7, 2.0)	
No	83 (78.3)	1	
Shared syringe/needle with another person			
Yes	117 (80.1)	1	
No	177 (74.1)	0.7 (0.4, 1.2), *p* = 0.2	1.779 (0.89, 3.57), *p* = 0.104
Number of sexual partners			
None or one	36 (73.5)	1	
Two or more	258 (76.6)	1.2 (0.6, 2.3)	
Had sexual intercourse with nonregular partner in the last 6 months			
Yes	135 (73.4%)	1	
No	159 (78.7%)	0.745 (0.47, 1.19), *p* = 0.22	
If the partner used condoms in the last sexual intercourse			
Yes	59 (66.3%)	1	
No	75 (79.8%)	0.49 (0.26, 0.97), *p* = 0.04	1.81 (0.91, 3.58), *p* = 0.09
Sexual partners were injecting drugs			
Yes	44 (69.8%)	1	
No	103 (75.7%)	0.742 (0.38, 1.44), *p* = 0.38	
If partners exchanged sex with other commodities			
Yes	123 (74.1%)	1	
No	171 (77.7%)	0.82 (0.51, 1.31), *p* = 0.41	

## Data Availability

The data used to support the findings of this study are available from the corresponding author upon request.
